# Reconnoitering correlation between human papillomavirus infection-induced vaginal microecological abnormality and squamous intraepithelial lesion (SIL) progression

**DOI:** 10.1186/s12905-023-02824-z

**Published:** 2024-01-02

**Authors:** Jiawei Li, Haihong Jin, Yongmei Sun, Chunhua Wang, Hongjuan Chen, Shan Gong, Li Jiang

**Affiliations:** 1 Department of Gynecology, Qinhuangdao First Hospital, 258 Wenhua Road, Haigang District, Qinhuangdao, Hebei 066099 China; 2 Department of Inspection Center, Qinhuangdao First Hospital, Qinhuangdao, Hebei 066099 China

**Keywords:** Vaginal microecology, High-risk human papillomavirus, Low-risk human papillomavirus, Low-grade squamous intraepithelial lesion, High-grade squamous intraepithelial lesion

## Abstract

**Objective:**

This study aims to investigate the relationship between abnormal vaginal microecology and human papillomavirus (HPV) infection, as well as the squamous intraepithelial lesions (SIL) progression.

**Methods:**

A total of 383 patients diagnosed with HPV infection in our hospital between March 2017 and February 2022 were selected as the experimental group. In addition, several volunteers (n = 898) who underwent physical examination during the same period were randomly selected as the control group. Subsequently, we conducted several investigations, such as HPV detection and gene typing, examined vaginal microecological imbalances, and performed cytological examinations to analyze the correlation between microecological changes, different types of HPV infection, and SIL progression.

**Results:**

HPV detection primarily included single and high-risk types of HPV infections. Moreover, significant disparities in the vaginal microecological environment between patients with persistent HPV infection and the control group, as well as patients with low-grade and high-grade SIL (LSIL and HSIL), were observed. The regression analysis revealed a correlation between LSIL and microflora density, diversity, bacteriological vaginosis (BV), vulvovaginal candidiasis (VVC), trichomonas vaginalis (TV), sialidase, as well as *Lactobacillus.* In addition, we identified an association between HSIL and pH, flora density, diversity, BV, VVC, candida vaginitis (CV), leukocyte esterase, catalase, and *Lactobacillus* levels.

**Conclusion:**

These findings revealed a significant association between abnormal vaginal microecology and both HPV infection and the SIL progression.

## Introduction

Cervical cancer has emerged as a prevalent malignancy, significantly contributing to increased global mortality among women [1, 2]. Typically, the pathophysiological etiology of cervical cancer is closely related to the oncogenic human papillomavirus (HPV) infection, playing an active role in cervical epithelial transformation [3, 4]. HPV infection and its persistence often lead to the development of squamous intraepithelial lesions (SIL), which may either resolve or persist and progress to a dreadful cancer [1]. Although specific HPV subtypes played a pivotal role in initiating and advancing cervical squamous cell carcinoma, previous reports indicated that HPV alone could be insufficient to induce malignant cervical transformation [4–7]. In addition, several reports demonstrated the correlation between various other factors and the onset and progression of SIL, including menstrual bleeding, multiple childbirths, extensive contraceptive use, multiple sexual partners, and smoking [8, 9].

In addition to the aforementioned significant factors, the vaginal microecological system plays a pivotal role in the development of HPV infection, thereby contributing to the onset of cervical cancer [10]. According to the principles of infection biology, several essential attributes for successful pathogen colonization and infection encompass a dynamic interplay involving the infecting microorganism, host factors, and the surrounding microecology [11]. Along this line, the highly complex microbial ecosystem of the vagina is influenced by numerous environmental, host-related factors, and ethnic factors [12]. Although the typical vaginal microflora in healthy women consists of bacteria from over 200 genera, this vaginal ecosystem is primarily characterized by the dominance of *Lactobacillus* spp. Notably, lactobacilli often provide robust protection against pathogenic infections in the vaginal milieu by generating substantial quantities of lactic acid, bacteriocins, and biosurfactants, thereby forming a critical barrier to mucosal invasion [2, 13]. In this context, the term “vaginal microbiota equilibrium” denotes a state of beneficial condition in which the quantity, diversity, and interplay among microorganisms are maintained within the vagina of the host. In addition to this defense system, several physicochemical changes frequently manifest in the vaginal microenvironment, resulting in histological alterations in the mucosa and cervical epithelium, exerting selective pressures on the microbiota [14–16]. Contrarily, the microbial diversity within the vagina, referring to the abundance of microbial species, is considered advantageous for vaginal health as it serves to curb the excessive proliferation of pathogenic microorganisms. An imbalance in the microbial composition can lead to undesirable disturbance in the vaginal microbiota, potentially resulting in infections and other health complications.

To this end, several other vaginal microbes, including *Gardnerella*, *Clostridium*, *Bacillus coelicolor*, *Microbacterium*, *Prevotella*, and *Mycoplasma*, have been identified. Nevertheless, these microbiota often exist in decreased proportions compared to *Lactobacillus*. These microbes associated with dysbiosis can lead to an unstable vaginal microecological environment, potentially contributing to various key risk factors for cervical cancer [17–20]. Moreover, the altered conditions may result in amplified levels of mucin-degrading enzymes, playing a role in degrading the mucous layer covering the epithelium and mucus of the cervix [21, 22]. Previous reports on HPV evasion or infection mechanisms suggested that microorganisms from the *Lachnospiraceae*, *Fusobacterium*, and *Gardnerella* genera could be associated with a higher degree of severity, potentially leading to precancerous lesions or cervical carcinogenesis [23]. Despite significant progress in understanding the underlying causes of cervical cancer and HPV infection, the relationship between HPV infection, SIL, and vaginal microecology remains to be systematically explored.

Motivated by these considerations, this study investigated the association between abnormal vaginal microecology and HPV infection, as well as the SIL progression. In this article, we examined a cohort of HPV-infected patients (n = 383) and assessed the status of cervical infection and genotype distribution. In addition, a comparative analysis of vaginal microecology among several groups was conducted, including HPV-negative individuals as the control treatment group, patients with HPV infections, persistently-infected individuals, and patients with cervical SIL. The comparative analysis not only allowed us to explore the correlation between HPV infection and SIL progression but also shed light on the concept of vaginal microecological dysbiosis. In summary, we firmly believe that establishing such a relationship will undeniably contribute to the development of innovative concepts and strategies for the prevention and treatment of cervical cancer in the future.

## Materials and methods

### General information

#### Subjects

A total of 383 patients who were diagnosed with HPV infection at our hospital between March 2017 and February 2022 were categorized as the experimental group. The selected patients were in the age range of 18 to 60, with an average age of 36.55 ± 4.21 years. Among them, 232 patients have reportedly given birth previously. In contrast, the control group of volunteers (n = 898) who tested negative for HPV at our hospital during the same period were recruited for this study. In the control group, 514 volunteers have reportedly given birth previously. Notably, no significant differences in the baseline data between the two (experimental and control) groups (P < 0.05) were evident. It should be noted that the study was approved by the Research Ethics Committee of our hospital. A written informed consent was provided by all participants before collecting the data for sampling.

#### Infection status

The infection status was classified as follows. The patients or individuals diagnosed with HPV at intervals exceeding 6 months were denoted as an HPV persistent infection group. Briefly, patients who tested positive for the same HPV subtype at least twice for a minimum of 6 months were classified as possessing HPV persistent infection. In contrast, patients with the HPV status turned negative and were categorized as a transient infection group.

#### Exclusion criteria

The exclusion criteria were set as follows: (1) Patients who were pregnant during the study period; (2) Female patients who were actively lactating for the newborn babies; (3) Patients who were diagnosed with other malignancies or history of any other malignant diseases; (4) Patients who were prescribed with the immunosuppressive diseases or under immunosuppressive therapy during the study; (5) Patients who were participants in an HPV vaccine clinical trial prior to the study. (6) Patients with a history of treatment for various cervical diseases.

### Methods

#### Cervical deoxyribose nucleic acid (DNA) extraction, HPV detection, and typing

Initially, the vaginal discharge samples were collected using sterile collection tubes equipped with an integrated sterile swab. Then, the surplus samples on the cervical surface were initially removed by gently wiping them with a disposable sterile dry cotton swab. Subsequently, the disposable HPV cervical brush was inserted deep into the cervical canal, rotated clockwise for three turns, and then gently placed into a preservation solution. Accordingly, the HPV genotyping was performed using polymerase chain reaction (PCR)-reverse dot blot hybridization (RDB) (Yaneng Biosciences Ltd., Shenzhen, China).

#### Vaginal microecological balance

The Vaginalis Five Enzyme-Linked Assay Kit (Zhengzhou Ruipu Biological Engineering Co., Ltd., Zhengzhou, PR China) was employed to determine the following parameters in accordance with the provided instructions: pH, flora density and diversity, candida vaginitis (CV), bacteriological vaginosis (BV), trichomonas vaginalis (TV), *Lactobacillus*, catalase, cleanliness, leukocyte esterase, sialidase, and other relevant indicators. The interpretation of results is as follows: The samples were distinguished as positive (+) if the result was a pale yellow or colorless well, signifying the presence of a small amount of lactobacilli. The samples were distinguished as weakly positive (±) if samples were pale red, suggesting a moderate quantity of lactobacilli. A negative result (−) was indicated for the samples with red or purple-red wells, denoting a large quantity of lactobacilli. In the case of Leukocyte esterase, the samples with blue color were presented as positive (+, ++, and +++). Meanwhile, a pale blue well was indicated as a weakly positive result (±). Contrarily, a well with no color or a light color was signified as a negative result (−). For Sialidase determination, the samples displayed red, purple-red, blue, brown, or black were considered positive (+). At the same time, a pale red well was characterized as a weakly positive result (±). Contrariwise, a well with no color or appearing orange-yellow was denoted as a negative result (−).

#### Liquid-based thin layer cytology test (TCT)

Briefly, the patients undergoing the TCT examination were positioned on the examination bed in the bladder stone position. After collecting the vaginal secretions, any excess secretions on the cervical surface were gently wiped away using a disposable sterile cotton swab. Subsequently, a single-use TCT sampler was inserted parallel into the cervical canal. After the lowest part of the brush bristles of the sampler brush head was exposed outside of the cervix, the sampler was gently secured, rotated clockwise for 3–5 turns, carefully removed, and gently placed into the preservation solution for subsequent TCT analysis.

### Outcomes and criteria

#### Distribution of HPV types (low- and high-risk)

A total of 25 HPV subtypes were detected among patients with HPV infection. Briefly, the HPV types of 6, 11, 40, 42, 43, 44, 81, 83, and 8 other subtypes were categorized as low-risk HPV types. Contrariwise, the HPV subtypes of 16, 18, 26, 31, 33, 35, 39, 45, 51, 52, 53, 56, 58, 59, 66, 68, 73, and 17 other subtypes were categorized as high-risk HPV infection types.

#### Normal vaginal microecology

Typically, the vaginal microenvironment maintains a pH range of 3.8–4.5, along with a density and diversity of vaginal flora classified as II-III. The microscopic examination under an oil lens revealed the presence of bacteria, particularly lactic acid bacteria. However, no significant amounts of white blood cells, fungi, or Trichomonas were observed.

#### Vaginal dysbiosis

The presence of significant amounts of fungi and Trichomonas has become apparent in cases of dysbiosis within the vaginal microenvironment at the intravaginal pH of > 4.5. Furthermore, the changes in the flora density, diversity, dominant bacterial species, and white blood cell counts were observed. Notably, the clinical indicators of vaginal congestion and edema, such as a drench-like discharge, were observed under an oil lens, confirming the diagnosis of Vulvovaginal Candidiasis (VVC). The diagnosis of VVC was further supported by the presence of Pseudomonas spores, blastospores, or hyphae. Bacterial Vaginosis (BV) was diagnosed based on the Nugent score, in which a score of ≥ 7 could indicate the presence of BV. Trichomonas Vaginitis (TV) was diagnosed with the symptoms, including the presence of thin, purulent, yellow-green, foamy discharge with an unpleasant odor, often accompanied by burning, pain, and discomfort during intercourse. Trichomonas in the vaginal samples were visible under an oil microscope. Aerobic vaginosis (AV) was diagnosed using a microscope under an oil lens. In addition, the dominant bacteria, such as gram-positive bacilli, cocci, or streptococci, were identified after culture. The reactivity of vaginal samples with the corresponding specific substrate was determined, and the activities of catalase, leukocyte esterase, and sialidase were assessed, following the manufacturer’s instructions of the Vaginitis Quintuple Test Kit (Zhengzhou Ruipu Biological Engineering Co., Ltd., Zhengzhou, PR China).

#### Liquid-based TCT results

The cytology test results were accurately interpreted by the specialized pathologists, referencing The Bethesda System (TBS). The dichotomous classification proposed by the World Health Organization (WHO) in 2014 was employed, distinguishing between low-grade SIL (LSIL) and high-grade SIL (HSIL). Notably, no discernible pathological changes were observed, signifying the absence of intraepithelial lesions and malignant cells. This outcome could correspond to a diagnosis of “negative for intraepithelial lesion or malignancy” (NILM).

### Statistical analysis

The experimental data were presented in terms of frequency (percentage, n, %). The data between the groups were compared using the χ² test and analyzed by the influencing factors logistic regression model, considering *P* < 0.05 statistically significant.

## Results

### Data show differential characteristics of HPV Infection and genotype distribution

Table 1 presents a comprehensive overview of the HPV infection status, in which different HPV subtypes are categorized. Among all the recruited subjects with HPV infection, there were 289 cases with a single subtype of infection, 57 cases with two subtypes, 31 cases with three subtypes, and 6 cases with four subtypes. Moreover, 74 patients were infected with a low-risk type (LR-HPV), 298 with a high-risk type (HR-HPV), and 11 with a combination of high- and low-risk types.

**Table 1:** A summary of 383 patients displays differential HPV infection status

HPV genotype	Single- infection	Double- infection	Triple- infection	Quadruple- infection	Total (n)
LR-HPV	57	7	9	1	74
HR-HPV	232	44	18	4	298
Mixed HPV type	0	6	4	1	11
Total (n)	289	57	31	6	383

Further, the frequency distribution of subtypes was examined in the case of patients infected with both high- and low-risk types of HPV. The most commonly detected high-risk HPV subtypes included HPV16, HPV52, and HPV58, accounting for 104 (27.15%), 63 (16.45%), and 48 (12.54%) cases, respectively. Table 2 shows a detailed breakdown of subtype frequency distribution.

**Table 2:** A summary shows the distribution of HPV genotypes in 383 HPV-positive patients

HPV genotype	Single-infection (n = 289)	Co-infection (n = 94)	Total (n)
HPV6	9 (2.34)	4 (1.04)	13
HPV11	5 (1.31)	3 (0.78)	8
HPV40	2 (0.52)	1 (0.26)	3
HPV42	22 (5.74)	4 (1.04)	26
HPV43	9 (2.34)	5 (1.31)	14
HPV44	11 (2.87)	3 (0.78)	14
HPV81	11 (2.87)	4 (1.04)	15
HPV83	1 (0.26)	1 (0.26)	2
HPV16	89 (23.23)	15 (3.92)	104
HPV18	11 (2.87)	4 (1.04)	15
HPV26	1 (0.26)	1 (0.26)	2
HPV31	17 (4.44)	3 (0.78)	20
HPV33	17 (4.44)	5 (1.31)	22
HPV35	13 (3.39)	3 (0.78)	16
HPV39	21 (5.48)	3 (0.78)	24
HPV45	1 (0.26)	1 (0.26)	2
HPV51	22 (5.74)	4 (1.04)	26
HPV52	55 (14.36)	8 (2.09)	63
HPV53	34 (8.89)	5 (1.31)	39
HPV56	25 (6.53)	4 (1.04)	29
HPV58	42 (10.97)	6 (1.57)	48
HPV59	7 (1.83)	1 (0.26)	8
HPV66	16 (4.44)	3 (0.78)	19
HPV68	17 (4.44)	2 (0.52)	19
HPV73	2 (0.52)	1 (0.26)	3

### Different HPV Infection groups show altered vaginal microenvironments

The correlation between HPV infection status and the resultant vaginal microecological system was examined. To investigate the variations in the vaginal microecological system, the levels of pH, BV, AV, VVC, TV, CV, leukocyte esterase, sialidase, catalase, and *Lactobacillus* were initially observed and subsequently compared among different HPV infection groups. It should be noted that all the mentioned indicators exhibited statistically significant differences (P < 0.05) while comparing the patients with HPV transient infection, HPV persistent infection, and the HPV-negative population groups. However, no significant difference was observed in terms of the density and diversity of bacterial flora among patients with HPV transient infection, HPV persistent infection, and the HPV-negative population groups (P > 0.05, Table 3).

**Table 3:** Data indicate changes in various factors in the vaginal microenvironment between different HPV infection groups

Variables	HPV-negative 898 cases	Transient HPV infection (n = 288)	Persistent HPV infection (n = 95)	*P*
pH				
Normal	172 (19.15)	27 (9.38)	5 (5.26)	0.037
Abnormal	726 (80.85)	252 (90.62)	90 (94.74)	
**Microbiome density**				
Normal	536 (59.69)	167 (57.98)	59 (62.11)	0.117
Abnormal	362 (40.31)	121 (42.02)	36 (37.89)	
**Microbiome diversity**				
Normal	592 (65.92)	156 (54.17)	58 (61.05)	0.095
Abnormal	306 (34.08)	132 (45.83)	37 (38.95)	
**BV**				
Positive	267 (29.73)	135 (46.88)	65 (68.42)	< 0.001
Negative	631 (70.27)	153 (53.12)	30 (31.58)	
**AV**				
Positive	215 (23.94)	116 (41.13)	69 (72.63)	< 0.001
Negative	683 (76.06)	172 (58.87)	26 (27.37)	
**VVC**				
Positive	152 (16.93)	109 (37.85)	57 (60.0)	< 0.001
Negative	746 (83.07)	179 (62.15)	38 (40.0)	
**TV**				
Positive	119 (13.26)	89 (30.91)	56 (58.95)	< 0.001
Negative	779 (86.74)	199 (69.09)	39 (41.05)	
**CV**				
Positive	177 (19.71)	84 (29.17)	61 (64.21)	< 0.001
Negative	721 (80.29)	204 (70.83)	34 (35.79)	
**Leukocyte esterase**				
Positive	699 (77.84)	225 (78.13)	81 (85.26)	0.039
Negative	199 (22.16)	63 (21.87)	14 (14.74)	
**Sialidase**				
Positive	6 (0.67)	3 (1.04)	3 (3.16)	0.047
Negative	892 (99.33)	285 (98.96)	92 (96.84)	
**Catalase**				
Positive	362 (40.31)	158 (54.86)	63 (66.31)	0.029
Negative	563 (59.69)	130 (45.14)	32 (33.69)	
***Lactobacillus***				
Positive	405 (45.11)	128 (44.44)	52 (54.74)	0.034
Negative	493 (54.89)	160 (55.56)	43 (45.26)	

### Multivariate logistic regression analysis presents an association between the HR-HPV Infection and the reported factors

Furthermore, the correlation between HR-HPV persistent infection and various other factors related to the vaginal environment was explored. A multivariate logistic regression analysis was performed to assess the exposures of HR-HPV transient and HR-HPV persistent infections. The regression analysis results demonstrated a significant correlation between HR-HPV transient infection and the reported factors, such as pH level, microbiome density, and diversity, BV, AV, VVC, CV, sialidase, and *Lactobacillus* (P < 0.05). Furthermore, HR-HPV persistent infection exhibited a significant association with pH level, microbiome diversity, BV, AV, VVC, TV, CV, leukocyte esterase, sialidase, and *Lactobacillus* levels (P < 0.05, Table 4).

**Table 4:** Multivariate logistic regression analysis indicates the risk of transient/persistent HPV infection

Variables	Transient HPV infection		Persistent HPV infection	
	OR	*P*	OR	*P*
pH	0.965 (0.696 ~ 1.582)	0.022	0.995 (0.548 ~ 1.791)	0.015
Microbiome density	1.298 (0.553 ~ 1.989)	0.004	1.319 (0.855 ~ 1.847)	0.098
Microbiome diversity	0.963 (0.795 ~ 1.167)	0.016	0.915 (0.637 ~ 1.275)	0.016
BV	2.198 (1.025 ~ 3.874)	0.001	1.689 (0.497 ~ 2.973)	0.004
AV	1.372 (0.837 ~ 1.286)	0.017	0.993(0.748 ~ 1.305)	0.045
VVC	1.913 (0.732 ~ 2.587)	0.008	0.995 (0.611 ~ 1.894)	0.039
TV	1.176 (0.938 ~ 1.478)	0.162	1.319 (0.855 ~ 1.847)	0.018
CV	1.158 (0.465 ~ 2.164)	0.031	2.187 (0.652 ~ 4.923)	0.011
Leukocyte esterase	1.028 (0.984 ~ 1.426)	0.116	1.783 (0.852 ~ 3.733)	< 0.001
Sialidase	0.965 (0.691 ~ 1.756)	0.017	1.217 (0.775 ~ 2.351)	0.023
Catalase	1.298 (1.053 ~ 1.589)	0.084	1.672 (0.653 ~ 3.019)	0.097
*Lactobacillus*	1.327 (0.795 ~ 1.826)	0.016	2.091 (0.859 ~ 4.004)	0.000

### Patients with different SIL groups show vaginal microecological changes

Table 5 explicitly illustrates the levels of various related factors within the vaginal microecological system. It was observed from the results that no significant differences (P < 0.05) in the microbiome density and diversity, BV, AV, VVC, TV, CV, leukocyte esterase, sialidase, catalase, as well as *Lactobacillus* levels within the vaginal microecological system were evident while comparing LSIL and HSIL patients to NILM patients.

**Table 5:** Different SIL groups display changes in microenvironment factors

Variables	NILM (n = 317)	LSIL (n = 41)	HSIL (n = 25)	*P*
**pH**				
Normal	26 (8.2)	4 (9.75)	2 (8.0)	0.226
Abnormal	291 (91.8)	35 (85.36)	23 (92.0)	
**Microbiome density**				
Normal	199 (62.78)	19 (46.34)	8 (32.0)	< 0.001
Abnormal	118 (37.22)	25 (53.66)	17 (68.0)	
**Microbiome diversity**				
Normal	182 (57.41)	22 (53.66)	10 (40.0)	0.035
Abnormal	135 (42.59)	19 (46.34)	15 (60.0)	
**BV**				
Positive	165 (52.05)	18 (43.9)	17 (64.0)	0.037
Negative	152 (47.95)	23 (56.1)	8 (36.0)	
**AV**				
Positive	151 (47.63)	19 (46.33)	15 (60.0)	0.029
Negative	166 (52.37)	22 (53.67)	10 (40.0)	
**VVC**				
Positive	135 (42.59)	18 (43.9)	13 (52.0)	0.046
Negative	182 (57.41)	23 (56.1)	12 (48.0)	
**TV**				
Positive	114 (35.96)	17 (41.46)	14 (56.0)	0.021
Negative	203 (64.04)	24 (58.54)	11 (44.0)	
**CV**				
Positive	120 (37.85)	13 (31.7)	12 (48.0)	0.045
Negative	197 (62.15)	28 (68.3)	13 (52.0)	
**Leukocyte esterase**				
Positive	250 (78.86)	34 (82.91)	22 (88.0)	0.039
Negative	67 (21.14)	7 (17.09)	3 (12.0)	
**Sialidase**				
Positive	1 (0.32)	2 (4.88)	3 (12.0)	< 0.001
Negative	316 (99.68)	39 (95.12)	22 (88.0)	
**Catalase**				
Positive	182 (57.41)	20 (48.78)	19 (76.0)	< 0.001
Negative	125 (42.59)	21 (51.22)	6 (34.0)	
***Lactobacillus***				
Positive	145 (45.74)	18 (43.9)	17 (68.0)	< 0.001
Negative	172 (54.26)	23 (56.1)	8 (32.0)	

### Multivariate logistic regression analysis shows LSIL and HSIL incidence risks

The multivariate logistic regression analysis revealed a significant association between the onset of LSIL and other reported factors, including microbiome density and diversity, BV, VVC, TV, sialidase, and *Lactobacillus* (P < 0.05). Similarly, the HSIL onset was significantly associated with the vaginal pH levels, microbiome density and diversity, BV, VVC, CV, leukocyte esterase, catalase, and *Lactobacillus* levels (P < 0.05, Table 6).

**Table 6:** Multivariate logistic regression analysis shows the LSIL/HSIL risks

	LSIL		HSIL	
	OR	*P*	OR	*P*
pH	0.718 (0.318–1.138)	0.112	2.983 (1.781 ~ 3.684)	0.012
Microbiome density	0.918 (0.527–1.098)	0.039	1.728 (0.668 ~ 2.951)	0.033
Microbiome diversity	1.012 (0.819 ~ 1.319)	0.024	1.137 (0.831 ~ 1.489)	0.004
BV	2.117 (1.003 ~ 3.418)	< 0.001	1.376 (0.693 ~ 1.792)	0.022
AV	1.263 (1.041 ~ 1.538)	0.183	0.998 (0.771 ~ 1.273)	0.173
VVC	1.982 (0.951 ~ 3.217)	0.019	2.114 (0.894 ~ 3.759)	0.012
TV	1.737 (0.673 ~ 4.696)	0.005	1.694 (1.071 ~ 2.398)	0.084
CV	1.026 (0.971 ~ 1.075)	0.215	1.787 (0.684 ~ 3.006)	0.006
Leukocyte esterase	0.895 (0.673 ~ 1.128)	0.104	1.935 (0.812 ~ 4.078)	< 0.001
Sialidase	1.784 (1.041 ~ 3.797)	0.006	1.047 (0.839 ~ 1.307)	0.097
Catalase	1.227 (0.902 ~ 1.784)	0.163	1.698 (0.485 ~ 2.709)	0.027
*Lactobacillus*	2.058 (0.896 ~ 3.019)	0.001	2.091 (0.934 ~ 3.078)	0.000

## Discussion

In our current study, it was initially recognized that high-risk HPV genotypes, including HPV16, HPV52, and HPV58, were the most prevalent subtypes among the recruited patients with high-risk HPV infections. Secondly, significant differences in vaginal microecological parameters were observed, such as pH, BV, AV, VVC, TV, CV, leukocyte esterase, sialidase, catalase, and *Lactobacillus* levels among the HPV transient infection, persistent infection, and HPV-negative groups of patients. Nevertheless, the bacterial flora diversity in the vaginal microenvironment showed no significant variations among these HPV transient infection, persistent infection, and HPV-negative groups of patients. Furthermore, the multivariate regression analysis unveiled a substantial correlation between HR-HPV infections and specific vaginal parameters, as well as the association between the LSIL and HSIL incidence rates and microbiome-related factors. These findings suggested that the impact of HPV infections on the vaginal microecology could pose potential implications for cervical lesions.

Previous reports indicated that HPV could substantially play a role in the development of cervical pre-invasive and invasive diseases, including cervical cancer. Although several HPV subtypes of over 200 have been identified so far, only a few have been found to possess the oncogenic potential [24]. Among them, HPV-16 and HPV-18 have emerged as the most closely related subtypes to developing highly invasive cancer, accounting for approximately 65–75% of cervical cancer cases. Further, it has been increasingly recognized that these HPV-related persistent infections and other high-risk oncogenic HPV subtypes could result in precancerous lesions. Accordingly, the current study demonstrated that the major proportion of patients was infected with a single HPV subtype. Meanwhile, high-risk types (HR-HPV) accounted for a relatively more significant proportion of patients over LR-HPV. In this context, the major subtypes, including HPV16, HPV52, and HPV58, showed the highest frequency of infection [25]. Typically, most of the viruses, including HPV infection, and their debris can be cleared by the immune system, resulting in the absence of any clinical symptoms in patients. However, there exist some infected cases of women who might be developing a persistent infection and progressing to LSIL or HSIL and cervical cancer [26]. Moreover, the resultant persistent HPV infection may be associated with the recurrence of cervical lesions [27, 28]. In this study, a total of 288 cases of HPV-infected individuals possessed the transient infection, while 95 cases with persistent HPV infection accounted for approximately 25% of HPV-infected patients.

In this vein, this study highlighted the complicated relationship between HPV severity and changes in the vaginal microecological environment. Evidently, an imbalance in the vaginal microbiota can substantially activate the inflammation within the cervical environment. Subsequently, the resultant inflammatory responses may lead to epithelial damage in the cervix, thereby increasing susceptibility to cervical dysplasia. Besides, the vaginal microbial communities balancing the microecology can significantly influence the immune milieu within the cervical region. Accordingly, a well-maintained equilibrium of the vaginal microbiota is conservatively associated with the immune system stability in the body. Conversely, the microbial imbalance may precipitate immune system dysfunction, rendering the cervix susceptible to HPV infection. Typically, among the diverse communities of microbiota, *Lactobacillus* spp. play a crucial role in a healthy premenopausal vaginal environment towards maintaining a low acidic pH, serving as the first line of defense against pathogens for vaginal health [29]. Furthermore, these bacterial communities produce various protective polypeptides and metabolites that inhibit bacterial growth and disrupt biofilms. In the cases of weakened immunity or increased foreign pathogens, several other kinds of bacteria may replace *Lactobacillus* spp. as the dominant species, significantly elevating the risk of HPV infection and reducing the ability to clear HPV. Leukocyte esterase, typically released by leukocytes upon rupture, serves as an indicator of inflammatory responses, reflecting vaginal mucosal damage or the presence of inflammatory reactions. Several reports indicated that leukocyte esterase abnormalities would result in SIL in individuals infected with the HPV16 subtype. In a case, it was reported that the release of catalase that could break down pathogen-killing intracellular hydrogen peroxide could increase the risk of HPV infection or delay HPV clearance [30]. In addition, the abnormal sialidase levels could exacerbate disturbances in the vaginal microecological environment, creating a vicious cycle and heightening the risk of HPV infection [30].

Bacterial vaginosis (BV), often caused by *Gardnerella vaginalis*, is a prevalent infectious disease among women of reproductive age. A meta-analysis of cross-sectional studies indicated that the presence of BV could be associated with high rates of HPV infection [31]. Several pieces of evidence suggested that infections were more likely to persist in individuals with alterations in their microbiota state. In one case, the prevalence of BV was found to be 11% in women with persistent HR-HPV infection, in which the HR-HPV infection was cleared in only 5% of these individuals [32]. Similarly, King and colleagues reported that patients with delayed HPV clearance among 76 women were diagnosed with BV [33]. Consequently, it has been increasingly recognized that there exists a strong association between HPV infection and BV. However, the relationship between HPV infection and BV, as well as the association of BV with SIL, remains inconclusive.

Typically, the characteristic vaginal pH in a healthy state is < 4.5. An increase in the pH value is an indicator of vaginal microbial dysbiosis. In the case of dysbiosis, the vaginal microenvironment often exhibits an elevated pH of > 4.5, accompanied by a decrease in the levels of lactic acid bacteria or the occurrence of bacterial vaginosis (BV). Notably, most vulvovaginal candidiasis (VVC) patients exhibit a vaginal pH value of ≤ 4.5, which can potentially introduce the reported factors in the study. Nevertheless, the established relationship between HPV infection and the VVC condition remains a subject of controversy in current research. Several reports suggested that VVC might lead to reduced clearance of HPV infection, while others indicated that VVC might offer protection against HPV infection [34]. On the other hand, trichomoniasis (TV), one of the most common sexually transmitted infections caused by Trichomonas vaginalis, is characterized by damage to the vaginal mucosal epithelium, glycogen depletion, decreased lactate synthesis, and an increased intravaginal pH, increasing the risk of HPV infection. Previous studies indicated that TV infection was not significantly associated with HPV. AV and BV conditions share some similarities in terms of increased vaginal secretions and elevated vaginal pH. However, an increase in these conditions is more pronounced in AV than in BV. Moreover, AV patients may specifically present with vaginal redness or even erosions and ulcers. In our study, we observed significant changes in the levels of pH, BV, AV, VVC, TV, CV, leukocyte esterase, sialidase, catalase, and Lactobacillus in the vaginal microecological system while compared with HPV transient infection, HPV persistent infection, and HPV-negative population groups. Nevertheless, no significant differences were noted in the density and diversity of the microbiome community in the vaginal microecological system. Furthermore, it was observed that patients with persistent HPV infection exhibited more significant changes in the aforementioned aspects compared to those with HPV transient infection (P < 0.05). Reportedly, HPV-infected patients often exhibited a more diverse flora with a decreased proportion of lactic acid bacteria [35]. These findings suggested that open abdominal radical hysterectomy yielded better results than laparoscopic radical hysterectomy [36, 37].

Eventually, we investigated the key manifestations of vaginal microecology in the recruited patients with varying degrees of cervical SIL. In a case, researchers indicated that patients with LSIL exhibited a 2-fold increase in the rate of lactic acid bacteria depletion. At the same time, patients with HSIL showed an increase by 3-fold compared to a healthy HPV-negative control group of patients [38]. These shreds of evidence suggested that interventions in vaginal microecology might offer a potentially effective means of preventing the development of precancerous lesions. Our current study observed more significant vaginal microecological differences in LSIL and HSIL patients compared to NILM patients. In the current study, we identified significant differences in microbiome density and diversity, BV, AV, VVC, TV, CV, leukocyte esterase, sialidase, catalase, as well as Lactobacillus levels. In addition, the regression analysis revealed no significant differences in LSIL incidence rate and microbiome density between LSIL and HSIL patients. However, sialidase and *Lactobacillus* levels were significantly correlated between LSIL and HSIL patients (P < 0.05). The HSIL onset was significantly associated with pH, microbiome density and diversity, BV, VVC, CV, leukocyte esterase, catalase, and *Lactobacillus* levels (P < 0.05). These findings suggested that variations in the vaginal microecology could play critical roles in the differential progression of cervical SIL. In this context, several aforementioned factors, such as pH, TV, CV, leukocyte esterase, and catalase, among others, might be critical in the progression from LSIL to HSIL. Despite establishing the significant relationship between HPV infection and the vaginal microecological environment, our study has some limitations. For instance, the comprehensive relationship between various HPV types and their specific characteristics, along with vaginal microecology, remains unexplored, which will be the focus of our future research.

## Conclusion

In summary, this research has revealed the crucial role of vaginal microecology in both HPV infection and the SIL progression. A deeper understanding of these relationships could pave the way for innovative strategies aimed at preventing and treating cervical cancer, ultimately improving the health outcomes of women. Moreover, it should be noted that maintaining a balanced vaginal microenvironment might emerge as a crucial factor in reducing the risk of HPV persistence and the development of SIL. However, the major limitation of our study included the relatively small number of patients involved in a single medical institution, with a relatively small sample size and the potential for bias, requiring a large sample of patients to validate the results. In addition, the lack of comprehensive information on patients in both groups is another limitation. Accordingly, studies in the near future should aim to quantify this phenomenon, possibly by employing next-generation sequencing (NGS) approaches to assess microbiome diversity. Therefore, further research involving larger and more diverse populations is warranted to validate and expand upon our findings.

## Data Availability

The table supporting the results of this study is included in the article, and the original datasets are available from the first author or corresponding author upon request.
